# Comparative efficacy of closed reduction versus open reduction with pelvic osteotomy for developmental dysplasia of the hip in children aged 18–24 months: A retrospective cohort study

**DOI:** 10.1371/journal.pone.0324995

**Published:** 2025-05-20

**Authors:** Qian Sun, Wenquan Cai, Guoxin Nan

**Affiliations:** 1 Department of Orthopedics, Children’s Hospital of Soochow University, Suzhou, Jiangsu Province, China; 2 Department of Orthopaedics Children’s Hospital of Chongqing Medical University, National Clinical Research Center for Child Health and Disorders, Ministry of Education Key Laboratory of Child Development and Disorders, Chongqing, China; 3 Chongqing Key Laboratory of Structural Birth Defect and Reconstruction, Chongqing, China; 4 Dongguan Children’s Hospital, Dongguan, Guangdong Province, China; 5 Dongguan Eighth People’s Hospital, Dongguan, Guangdong Province, China; South Valley University Faculty of Medicine, EGYPT

## Abstract

**Background:**

Optimal treatment for developmental dysplasia of the hip (DDH) in 18–24-month-old children is debated. This study compares closed reduction (CR) to open reduction with pelvic osteotomy (ORPO) to determine efficacy and complications.

**Materials and methods:**

Data from 97 patients (131 hips) undergoing CR or ORPO (June 2012 to September 2019) were analyzed. Pre- and postoperative measures including acetabular index (AI), International Hip Dysplasia Institute (IHDI) grade, center-edge angle (CEA), Severin grades, Mckay criteria and avascular necrosis (AVN) were assessed. Statistical analysis compared outcomes and complications.

**Results:**

Of 131 hips, 101 underwent CR and 30 ORPO. Preoperative characteristics and radiographic outcomes did not significantly differ. Postoperative AI (CR: 25.2 ± 5.3°, ORPO: 24.3 ± 6.1°, *P* = 0.441) and CEA (CR: 25.6 ± 11.5°, ORPO: 29.2 ± 16.5°, *P* = 0.263) showed no significant differences. Satisfactory Severin grades were achieved in 58.4% (CR) and 56.6% (ORPO), *P = *1.000. Mckay grade II and above were observed in 66.3% of CR group and 66.7% of ORPO group, *P = *1.000. AVN above type Ⅱ incidence was 18.9% (CR) and 33.3% (ORPO), *P = *0.131. After using multiple linear regression and logistic regression to control confounding factors, we came to the same outcome. No significant differences were observed in postoperative AI, CEA, Severin grade, Mckay grade or AVN. CR had significantly lower total hospitalization costs. Among the CR group, 19 hips (18.8%) underwent secondary surgery. And their postoperative outcomes were comparable to those in the ORPO group.

**Conclusion:**

Closed reduction with spica cast immobilization is a viable treatment option for DDH in 18–24-month-olds, with close monitoring. Prompt consideration of secondary surgery is advised for residual acetabular dysplasia

## Introduction

Developmental dysplasia of the hip (DDH) is the most common malformation of the hip joint characterized by an inappropriate anatomical relationship between the acetabulum and the femoral head [1]. Early diagnosis and intervention are widely acknowledged as imperative for optimal outcomes [2]. The primary principle of treatment is to achieve concentric reduction. For those late-detected cases, the obstacles to reduction become more difficult to overcome, and the acetabular development is less predictable [3]. Unfortunately, a considerable number of children miss timely screening, thus missing the opportunity for optimal treatment. Treatment decisions often hinge on patient age. For patients younger than 6 months, Pavlik harness is the primary option. For children between 6–18 months old, closed reduction and spica immobilization (CR) was accepted by most scholars [4]. For patients over 2 years old, open reduction and pelvic osteotomy (ORPO) is the first choice. But controversy persists for those aged 18–24 months.

Considering the limited acetabular remodeling potential beyond 18 months, Salter et al recommend primary ORPO in children older than 18 months [5]. Murphy et al also tend to routinely perform an ORPO instead of closed reduction, assuming that acetabular anatomy will not normalize on its own [6]. However, ORPO has a higher potential for complications such as infection, iatrogenic impingement, displacement of the osteotomy site, sciatic nerve injury, premature closure of the triradiate cartilage, and risks associated with longer anesthesia time [7,8].

CR is generally recommended for patients older than six months [3] and generally achieve satisfactory outcomes despite the risk of re-dislocation, avascular necrosis (AVN), and residual acetabular dysplasia (RAD). But for older children, some studies suggest poor outcomes with CR [9,10], while others have also reported satisfactory efficacy in patients over 18 months [11–13].

Over the past 12 years, our team has performed CR in children aged 18–24 months, achieving outcomes comparable with ORPO. This study retrospectively compares the efficacy and complication risks of both approaches, hypothesizing that CR offers similar clinical and radiographic outcomes to ORPO with a lower incidence of surgical complications and reduced healthcare costs.

## Materials and methods

This study was reviewed and approved by the Institutional Review Board of Children's Hospital of Chongqing Medical University with the approval number: 2021–363, dated November 20^th^ 2021. Due to the retrospective nature of the study, the Institutional Review Board of Children's Hospital of Chongqing Medical University waived the need of obtaining informed consent. All procedures in our research, including but not limited to data collection and statistical analysis, were performed in accordance with the relevant guidelines and regulations. The data used in this study were accessed for research purposes from 12 December 2021–18 March 2022. All authors did not have access to information that could identify individual participants during or after data collection

We retrospectively reviewed the medical records of 97 patients (131 hips) who underwent CR or ORPO in our hospital from June 2012 to September 2019. The inclusion criteria were: 1) At least 24 months of post operative follow-up. 2) Diagnosed with DDH without any treatment before. 3) Underwent CR or ORPO. 4) The age at initial treatment was between 18–24 months. Patients with neuromuscular disease, history of septic hip, or other secondary dislocations were excluded. Patients whose follow-up time was less than 24 months or who had accepted other treatment were also excluded

All patients were treated by the same surgical team led by a senior surgeon. Two senior orthopedic surgeons performed all CR or ORPO surgeries. Treatment selection between CR and ORPO was based on the surgeon's preference after a thorough assessment to exclude contraindications. The decision-making process also considered input from the patient's guardians. All included patients met the same eligibility criteria, ensuring comparability between groups.

### Surgical procedure

CR was performed under general anesthesia without pre-operative traction. Adductor tenotomy was performed if the adductor was considered to hinder reduction. The hip was reduced by placing it in flexion position about 100 degrees and abducting it to the position of stability. Then, a hip spica cast was applied to maintain reduction. MRI was taken in 24 hours to evaluate the reduction of the femoral head. For most MRI showed good reduction, arthrogram was not performed regularly in this study. The cast was changed once after 6–8 weeks. During the interval of cast change, which was usually 18–20 hours, the patient's hip was kept in abduction and flexion position naturally. And an anteroposterior (AP) radiograph was taken after the cast had been changed. Plain radiography was taken regularly during follow-up. After 3–4 months of total immobilization, the cast was removed and an adjustable abduction orthosis was used for 6–9 months.

Open reduction was taken through the anterior approach, then structures obstructing reduction were released systematically. After the reduction of the femoral head, capsulorrhaphy was performed. In most cases, femoral shortening is performed as well. After concentric reduction, Salter or Pemberton pelvic osteotomies were done modify to the shape of the acetabulum. After surgery, the patient was immobilized in spica cast for 6–8weeks. Afterwards, the cast was removed and physical therapy was taken to improve the motion of hip. The patient was permitted to bear weight 3 months after surgery.

According to the means of treatment, those patients were divided into two groups: CR group and ORPO group.

### Data collection

The medical data of all patients were collected. General clinical attributes such as gender, age, operative side, duration of follow-up, were collected from the patient file for each hip. Anteroposterior (AP) pelvic radiographs of the hip were obtained pre- and postoperatively until the final follow-up. Preoperative acetabular index (AI) and International Hip Dysplasia Institute (IHDI) grade [14] were acquired from the preoperative AP X-ray. Based on the last follow-up radiographs, post-operative outcomes such as AI, center-edge angle (CEA), Severin grades [15] and AVN were obtained. Severin grades I and II were considered to indicate satisfactory outcomes, whereas Severin grade III and IV were considered unsatisfactory. AVN was evaluated according to the Bucholz-Ogden method [16]. We would like to regard type I AVN as normal hip for statistical analysis, because it is considered to be transient ischemia of the femoral head which can recover completely. McKay criteria [17] was used to assess the involved hip function at the last follow-up. Mckay grade I-II is regarded as satisfactory outcome.

All radiographic measurements were independently assessed by two senior pediatric orthopedic surgeons blinded to the treatment groups. Inter-rater reliability for continuous variables was evaluated using the intraclass correlation coefficient (ICC) under a two-way random-effects model with absolute agreement and single measurement. Postoperative AI and CEA measurements from 30 randomly selected cases per group (total n = 60) were analyzed, demonstrating excellent agreement (AI: ICC = 0.88, 95% CI: 0.82–0.92; CEA: ICC = 0.84, 95% CI: 0.76–0.89). For categorical variables, including IHDI classification, Severin grade, and AVN, two blinded pediatric orthopedic surgeons conducted independent assessments. Consensus ratings were adopted for agreements, while discrepancies were resolved by a third senior orthopedic surgeon. The McKay functional grade was assessed during routine clinical follow-ups by the attending orthopedic surgeon.

### Statistical analysis

Statistical analysis was performed using SPSS 25.0 (IBM, America). Continuous variables were analyzed by Kolmogorov-Smirnov test to assess for normality. Normally-distributed data were expressed as X ± S, and non-normally distributed data were expressed as median with interquartile range (Med [IQR]). The t test was used to compare the differences in AI and CEA among the two groups. The chi-squared test or Mann-Whitney U test was used to compare categorical and ordinal variables such as gender, side, IHDI grade, Severin grade, Mckay grade and AVN. A *P* value < 0.05 was considered significant for all statistical tests.

We use logistic regression and linear regression to analyze the association between surgical methods (CR or ORPO) and post-operative outcomes, either a base model or a model adjusted for confounding factors, which containing sex, age, side, preoperative AI, and IHDI grade. For continuous variables such as postoperative AI and CEA, multivariable linear regression was applied. Ordinal variables, including Severin grade, AVN, and McKay grade, were analyzed using ordinal logistic regression. Moreover, for clinical practicality, we categorized Severin grade, AVN, and McKay grade into satisfactory and unsatisfactory groups and performed binary logistic regression.

## Results

**Fig 1:**
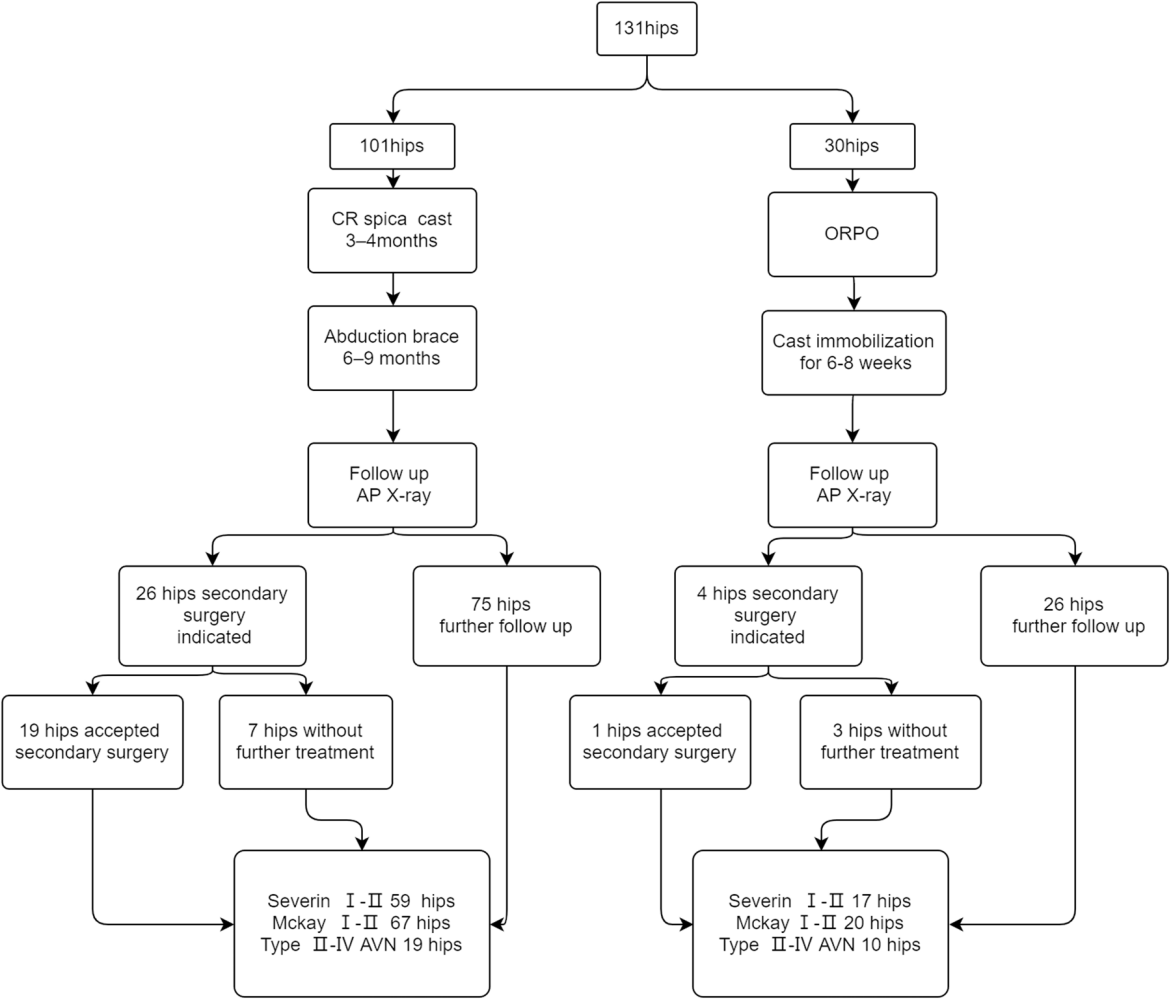
Clinical outcomes of the total 131 hips.

In total, 97 children (131 hips) with DDH received CR or ORPO. Initially 106 hips accepted CR, but 5 of them failed to achieve reduction and converted to ORPO immediately. Eventually, 101 hips underwent CR and 30 hips underwent ORPO (Fig 1). Among the CR group, 69 (68%) hips accepted adductor tenotomy. Mean preoperative AI was 37.7 ± 5.3° (range, 24.5°-55.0°). The follow-up time was 29[26,40] months (range, 24–95). In the ORPO group, 25 of 30 hips accepted a Salter osteotomy, while 5 hips had a Pemberton osteotomy. The femoral shortening was performed in 28 hips. Mean preoperative AI was 37.4 ± 5.6° (range, 27.6°-48.6°). The follow-up time was 38[24,49] months (range, 24–86). General clinical attributes and preoperative radiographic outcomes are listed in Table 1. There was no significant difference between two groups in age, gender, side, and IHDI grades.

**Table 1:** General clinical attributes and preoperative radiographic outcomes.

	total	CR Group	ORPO Group	*P* value
Number of hips	131(100%)	101(77.1%)	30(22.9)	
Sex				0.363
Female	113(86.3%)	89(88.1%)	24(80.0%)	
Male	18(13.7%)	12(11.9%)	6(20.0%)	
Side				0.838
Left	68(51.9%)	53(52.5%)	15(50.0%)	
Right	63(48.1%)	48(47.5%)	15(50%)	
Preoperative AI (degree)	37.6 ± 5.3	37.7 ± 5.3	37.4 ± 5.6	0.770
IHDI				
I	2(1.5%)	2(2.0%)	0	
II	13(9.9%)	13(12.9%)	0	
III	44(35.6)	34(33.7%)	10(33.3%)	0.084
IV	72(55.0)	52(51.5%)	20(66.7%)	
Follow-up time (months)		29[26,40]	38[24,49]	0.860

Normally distributed data were presented as mean ± standard deviation (M ± SD), while non-normally distributed data were expressed as median with interquartile range (Med [IQR]).

The postoperative outcomes are listed in Table 2. At the final follow-up, the mean postoperative AI of the CR group was 25.2 ± 5.3° for the CR group and 24.3 ± 6.1° for the ORPO group (*P = *0.441). The postoperative CEA of CR and ORPO group was 25.6 ± 11.5°and 29.2 ± 16.5 respectively (*P = *0.263). No significant differences were found in postoperative AI and CEA.

**Table 2:** Postoperative outcomes of different groups.

	CR group	ORPO group	*P* value
Postoperative- AI (degree)	25.2 ± 5.3	24.3 ± 6.1	0.441
Postoperative CEA (degree)	25.6 ± 11.5	29.2 ± 16.5	0.263
Total hospitalization cost (USD)	2160.87[1996.71,2602.70]	5195.09[4584.83, 5591.51]	<0.001
Severin grade			0.463
Ⅰ	40(39.6%)	9(30.0%)	
Ⅱ	19(18.8%)	8(26.7%)	
Ⅲ	30(29.7%)	8(26.7%)	
Ⅳ	12(11.9%)	5(16.7%)	
Satisfactory outcome	59(58.4%)	17(56.7%)	1.000
AVN			0.01
Normal	51(50.5%)	7(23.3%)	
Ⅰ	31(30.7%)	13(43.3%)	
Ⅱ	10(9.9%)	5(16.7%)	
Ⅲ	5(5.0%)	4(13.3%)	
Ⅳ	4(4.0%)	1(3.3%)	
AVN above type Ⅱ	19(18.8%)	10(33.3%)	0.131
Mckay grade			0.935
Ⅰ	45(44.6%)	14(46.7%)	
Ⅱ	22(21.8%)	6(20.0%)	
Ⅲ	19(18.8%)	5(16.7%)	
Ⅳ	15(14.9%)	5(16.7%)	
Satisfactory outcome	67(66.3%)	20(66.7%)	1.000

Normally distributed data were presented as mean ± standard deviation (M ± SD), while non-normally distributed data were expressed as median with interquartile range (Med [IQR]).

**Fig 2:**
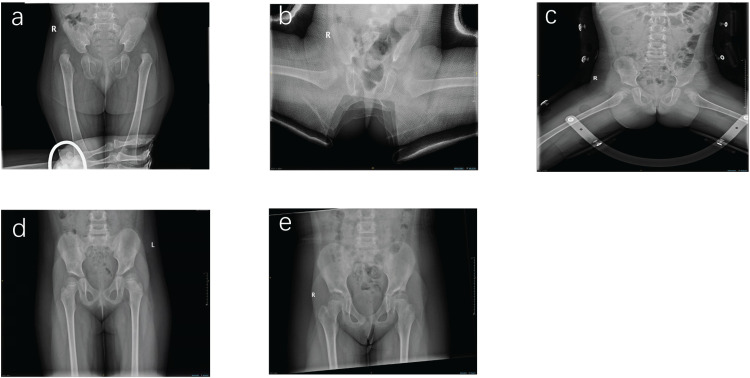
Radiological follow-up of a typical patient. The patient, a 20-month-old girl with bilateral DDH classified as IHDI grade IV. a) Pre-operative X-ray. b) Closed reduction and hip spica cast fixation were performed. c) After the cast was removed, an abduction orthosis was utilized. d) Pelvic radiographs after 24 months of follow-up. e) Pelvic radiographs after 80 months of follow-up.

Among the 101 hips in the CR group, 59 hips (58.4%) achieved Severin grade I or II, indicating satisfactory outcomes. In the ORPO group, 17 hips (56.6%) reached the same level. There was no significant difference in Severin grade between the two groups (*P = *1.000), suggesting comparable outcomes in terms of hip joint quality and residual dysplasia. Fig 2 shows the radiographic follow-up of a 20-month-old girl who underwent closed reduction, demonstrating a satisfactory outcome.

In CR group, 67 hips (66.3%) reached Mckay grade I or II, indicating satisfactory outcomes. In ORPO group, the number was 20 (66.7%). No statistic difference was found between those groups, indicating both treatment method could achieve comparable hip joint function

Among the CR group, AVN was detected in 60 hips (59.4%), with 19 hips (18.9%) classified as type II and above. In contrast, the ORPO group showed AVN in 20 hips (66.7%), including 10 hips (33.3%) with type II-IV AVN. A significant difference was observed in the incidence of AVN (*P = *0.01), primarily attributable to type I AVN. However, according to the Bucholz-Ogden method, type I AVN is considered unimportant as it may fully recover. The P-value for type II-IV AVN was 0.131, indicating no significant difference.

As shown in Table 3 and 4, linear regression and logistic regression were employed to control for confounding factors, including sex, age, side, preoperative AI, and IHDI grade. Multivariable** **linear regression revealed no significant differences in post-operative AI (Coefficient: -0.75, 95% CI: -3.30 to 1.33, *P = *0.410) and CEA (Coefficient: 0.109, 95% CI: -2.29 to 8.93, *P = *0.243) between the two surgical methods after adjustment. Similarly, when considering the ordinal outcomes Severin grade (OR: 1.18, 95% CI: 0.54 to 2.60, *P = *0.675) and McKay grade (OR: 0.90, 95% CI: 0.40–2.03, *P = *0.800), no significant differences were found through ordinal logistic regression. However, AVN demonstrated statistical significance (OR: 2.82, 95% CI: 1.25 to 6.37, *P = *0.013). Upon further investigation into the clinical implications of Severin, AVN, and McKay by examining Severin satisfactory (OR: 0.98, 95% CI: 0.40 to 2.41, *P = *0.965), AVN above type II (OR: 2.33, 95% CI: 0.83 to 6.57, *P = *0.108), and McKay satisfactory rates (OR: 0.80, 95% CI: 0.31 to 2.07, *P = *0.652), no statistical differences were observed. This suggests that, after controlling for confounding factors, the differences in post-operative outcomes between the two groups were not pronounced.

**Table 3:** Linear regression to investigate association between postoperative AI and CEA with different surgical methods.

	Unadjusted			Adjusted		
	Coefficient	95% CI	*P* value	Coefficient	95% CI	*P* value
Post-AI	-0.68	-3.13~1.37	0.44	-0.75	-3.30~1.33	0.410
CEA	0.12	-1.60~8.92	0.171	0.109	-2.29~8.93	0.243
Adjusted for sex, age, side, preoperative AI, and IHDI grade						

**Table 4:** Logistic regression to investigate association between postoperative AI and CEA with different surgical methods.

	Unadjusted			Adjusted		
	OR	95%CI	*P* value	OR	9%5 CI	*P* value
Severin Grade	1.31	0.63~2.73	0.465	1.18	0.54~2.60	0.675
Severin Satisfactory	1.07	0.47-2.45	0.865	0.98	0.40~2.41	0.965
AVN	2.61	1.24~5.47	0.011	2.82	1.25~6.37	0.013
AVN above type Ⅱ	2.16	0.87~5.35	0.097	2.33	0.83~6.57	0.108
Mckay Grade	0.97	0.46~2.06	0.934	0.90	0.40~2.03	0.800
Mckay Satisfactory	0.96	0.42~2.34	0.973	0.80	0.31~2.07	0.652
Adjusted for sex, age, side, preoperative AI, and IHDI grade.						

In the CR group, 19 hips experienced either re-dislocation (10 hips) or residual dysplasia (9 hips) during follow-up, necessitating further surgery involving pelvic osteotomy. Femoral shortening was implemented if necessary. At the latest follow-up, 9 hips (47.3%) achieved Severin grades I or II, and 9 hips reached McKay grade I or II. But 7 hips (36.8%) developed AVN of type II or above. These rates of satisfactory outcomes and AVN incidence are comparable to those observed in the ORPO group. However, due to the limited sample size, no statistical analysis was conducted. Additionally, one hip in the ORPO group accepted secondary surgery due to re-dislocation, resulting in Severin grade IV and type II AVN on the final anteroposterior X-ray. Table 5 showed the results of patients received secondary surgery in CR group. It should be noticed that, we recommended 26 hips in CR group and 4 hips in ORPO group to accept secondary surgery in total. But except for the cases mentioned above, the rest of them did not undergo further treatment in our institution or were lost of follow-up.

**Table 5:** Pre-and post- operative outcomes of hips accepted secondary surgery in CR group.

	Total 19 hips
Preoperative AI (degree)	37.57 ± 6.9
IHDI Classification	
I	0(0.0%)
II	1(5.3%)
III	5(26.3%)
IV	13(68.4%)
Postoperative AI (degree)	25.2 ± 6.8
Postoperative CEA (degree)	24.9 ± 14.4
Severin Grade	
I	3(15.8%)
II	6(31.6%)
III	6(31.6%)
IV	4(21.1%)
Satisfactory outcome	9(47.3%)
AVN Classification	
None	7(36.8%)
I	6(31.6%)
II	2(10.5%)
III	4(21.1%)
IV	0
AVN above type Ⅱ	6(36.8%)
Mckay Grade	
I	6(31.6%)
II	3(15.8%)
III	5(26.3%)
IV	5(26.3%)
Satisfactory outcome	9(47.3%)

We also gathered data on the total hospitalization costs for each patient In the CR group, cost was 2160.87[1996.71,2602.70] USD. Conversely, cost in the ORPO group was 5195.09[4584.83, 5591.51] USD. A significant difference in costs was observed (P < 0.001).

## Discussion

Studies have indicated a direct relationship between acetabular development and endochondral osteogenesis [18]. Biomechanical loading is thought to influence endochondral ossification, indicating that biomechanical factors may impact acetabular development [19,20]. In DDH patients, the normal biomechanical relationship between the acetabulum and the femoral head is disrupted, which would affect the ossification of the femoral head and the acetabulum. Therefore, the treatment of DDH follows a principle of providing stable, concentric reduction of the hip, facilitating early hip development [21,22].

Closed reduction plays an essential role during the process of DDH treatment through providing concentric reduction, with a high success rate and low complications, especially for young children. Most researchers tend to agree that age is a crucial factor in treatment selection [23]. The potential for acetabular remodeling diminishes over time. But there's ongoing debate regarding the duration of this potential. When the patient reaches the age limit when the acetabulum is less likely to remodel with time, the open reduction and pelvic osteotomy should be performed. Schwartz and Salter's study suggested that the cut-off time should be set at 18 months, which has been accepted by most researchers for many years [5,24]. However, several studies challenge this notion. Zamzam [25] proposed that acetabular remodeling continues for 3–4 years after closed reduction, with the most rapid changes occurring within the first six months. The study by Brougham et al [26] included 53 cases (60 hips) concluded that the acetabular remodeling stopped at a mean age of 5 years with some cases showing remodeling up to 8 years. Many other studies have reached similar conclusions [11,27,28], suggesting that acetabular remodeling may persist for up to 3 years after closed reduction, typically around the age of 4.

Thus, it's evident that the acetabulum retains remodeling potential in children aged 18–24 months, with closed reduction yielding satisfactory outcomes. In our study, the mean pre- and post-operative AI is 37.6 ± 5.32 ° and 25.2 ± 5.3 ° in CR group. And in the ORPO group, the mean pre- and post-operative AI is 37.7 ± 5.27 ° and 24.3 ± 6.1 °. There is no statistical difference in the Severin grade. Therefore, we posit that CR and ORPO have comparable effects on improving acetabular morphology and promoting acetabular remodeling. The research of Dai et al [29] also indicated that closed reduction was still a choice of management with close follow-up, which was analogous to ours. Long time follow up also found that closed reduction for patients over 18 months did not increase the incidence of total hip arthroplasty [30]. Noticeably, in the cases of CR group, most of whom were from Southwest China, failed closed reduction was rare (5 of 101 hips). However, in recent years, our team has observed a higher incidence of irreducible cases when performing the same procedure in South coastal regions of China. We speculate that regional differences and surgical team coordination may be contributing factors; however, we have no direct evidence to support this hypothesis, and further studies are needed for validation.

RAD stands out as one of the most common reasons for secondary operations in DDH cases. Advanced age at the time of closed reduction may correlate with a higher incidence of RAD [31]. The current diagnostic criteria for RAD remain a topic of debate. Some studies utilize AI to assess acetabular development [9,32], while the others prefer using Severin grade [6,33]. However, there's widespread agreement that surgical intervention is necessary to address RAD [34]. In our study, we opted to evaluate RAD using the Severin grade. The incidence of RAD (cases with Severin Ⅲ-Ⅳ) in both the CR and ORPO groups was 41.6% and 43.7%, respectively, with no significant difference noted. The same tendency could also be found in the McKay grade. The relatively lower satisfactory rates in Severin and McKay grades observed in both groups, compared to widely reported literature benchmarks, may be attributed to the older age range of our cohort (18–24 months) and the higher proportion of severe dysplasia (IHDI grades III-IV) at initial presentation. These factors are known to correlate with diminished remodeling potential and complex anatomical challenges, which may adversely impact functional and radiographic outcomes.

For patients who develop RAD following CR, secondary surgery often yields satisfactory results. Faciszewski et al [35] performed Pemberton's osteotomy on 42 children (52 hips) with RAD who had undergone CR previously, resulting in Severin Grade I or II outcomes for 51 hips. Chaker et al [36] used Salter's osteotomy to treat 31 children with RAD after closed reduction. Acceptable outcomes could be found in 88% of the case. However, for patients with failed open reduction, secondary surgery outcomes appear less favorable. A retrospective review was performed by Kershaw et al among 32 RAD patients who accepted secondary operation after the open reduction. More than half of the patients developed AVN, with over half of the patients developing AVN, and 11 cases reaching Severin grade III or worse.

In our research, 19 hips of the CR group accepted secondary surgery, 9(47.3%) hips reached satisfactory outcome, and 7(36.8%) hips developed AVN above type Ⅱ, which is similar to the CR group (56.6% of satisfactory Severin grade and 33.3% of AVN above type Ⅱ). Since no difference was found in the occurrence of RAD and secondary surgery usually achieves satisfactory outcome, we recommend that CR could be an effective option for DDH between 18–24 months. Other studies [12,23] also indicated similar point of view.

For those patients accepted CR, controversy remains about the time for a secondary operation if RAD was diagnosed. Most investigators recommend that the secondary surgery should be taken at 2–3 years after reduction, when the patient is about three to four years of age [24,37–39]. The other article [40] even indicates that the reconstructive surgery should be performed when the patient is 8–10 years old. However, we propose advancing this timeline to 6 months after the removal of the abduction orthosis. This decision is based on the understanding that acetabular remodeling occurs most rapidly during the initial 6 months after CR, followed by a slower pace of remodeling over the subsequent year [41]. By the time the orthosis is removed, approximately 15–18 months have passed since the initial closed reduction, during which the rate of acetabular remodeling has significantly diminished. At this point, most of the acetabular development has already occurred, and further prolonged follow-up may yield little additional benefit.

Schoenecker [12] and Zhang [31] both indicated that walking is detrimental to the acetabular development. Prolonged follow-up periods increase the likelihood of exposure to abnormal biomechanical burdens in RAD hips, potentially resulting in adverse outcomes. Shortening the follow-up period can mitigate the duration of abnormal walking mechanics and reduce the negative impact on acetabular remodeling. Additionally, prolonged follow-up and healthcare requirements may impose financial burdens on families, potentially leading to poor compliance or even treatment discontinuation. Therefore, we recommend that patients undergo further surgery if RAD persists 6 months after the removal of the orthosis, balancing the need for timely intervention with considerations of patient outcomes, financial implications, and treatment compliance.

The incidence of AVN varies widely between 15%-35% [9,11,31,42–44], which is caused by the different definitions of AVN and the timing of follow-up. The rate of AVN above type Ⅱ is 18.9% for the CR group and 33.3% for the ORPO group. Although no significant difference was found (*P = *0.131), the incidence of AVN is higher in the ORPO group. A similar result was mentioned in the article by Novais [44], where the incidence of AVN above type Ⅱ was 8% as well as 19% between the CR and OR groups, respectively. We suppose the higher rate of AVN in ORPO may be attributed to several factors. In ORPO, the femoral head is subjected to more extensive surgical manipulation, including capsule release, femoral head reduction and femoral osteotomy. These procedures, while necessary for optimal reduction, may also disrupt the femoral head's blood supply and impose mechanical stress on the hip. Furthermore, during the open reduction process, the femoral head is exposed to air, potentially contributing to compromised circulation and higher AVN risk.

It's important to note that AVN is considered an iatrogenic complication rather than a natural aspect of DDH [22,45]. While numerous factors have been identified as risk factors for AVN, the exact cause remains unclear. The prevailing assumption is that circulatory disorders and excessive pressure on the femoral head contribute to the development of AVN [46]. Therefore, we believe that gentle surgical techniques, adequate anesthesia, appropriate muscle relaxation, avoidance of forceful reduction, and preservation of blood supply to the femoral head are essential in reducing the incidence of AVN.

Apart from the advantages of being less traumatic and having a lower risk of anesthesia, our team also discovered that the cost for the CR group was significantly lower than that for the ORPO group. While few studies consider the cost of hospitalization, we believe it is of great importance, particularly for individuals in less developed regions. The cost of ORPO is prohibitively high, and parents may delay or even refuse treatment due to financial constraints, especially when the child's early symptoms do not affect daily life. This can often lead to irreversible consequences. Promoting closed reduction and close follow-up in economically disadvantaged areas to enhance patient compliance is of significant importance. Closed reduction also has its drawbacks, such as a prolonged treatment period, demanding nursing care, and the need for frequent follow-up visits. Most of our cases are from southwest part of China, the economic condition of which is not quite well developed. The care of a patient in a spica cast can impact the daily life of caregivers, but it is much more acceptable compared to the high costs of surgery. Additionally, traditional Chinese cultural beliefs often lead the majority of parents to be reluctant to accept invasive treatments. When making treatment decisions, the social context and the compliance of the patient's family should be taken into account.

However, our study has several limitations. Firstly, it is a retrospective study with a low level of evidence. Secondly, due to poor compliance with follow-up, many patients with mild condition were lost to follow-up shortly after treatment. Consequently, in our study, there is a higher proportion of IHDI III, IV cases and a higher incidence of complications compared to relevant literature. The limited sample size, especially in the ORPO group, may which may affect the generalizability of the findings. Lastly, further long-term follow-up is needed to assess the lasting efficacy of the treatments employed.

## Conclusion

In summary, our study found no statistically significant differences in postoperative AI, CEA, Severin grade or Mckay grade between the CR and ORPO groups. Similarly, there was no significant difference in the incidence of AVN of type II-IV between the two groups. These findings suggest that closed reduction is an effective treatment for DDH in children aged 18–24 months. For patients who undergo CR and develop residual acetabular dysplasia, satisfactory outcomes can often be achieved through secondary pelvic osteotomy.

Based on our findings, closed reduction with spica cast immobilization is a viable treatment option for DDH in children aged 18–24 months, showing comparable outcomes to ORPO with lower surgical morbidity. Close follow-up is essential, and secondary surgery should be considered if residual acetabular dysplasia persists.
